# Evaluation of Adverse Pregnancy Outcomes in Physicians Compared With Nonphysicians

**DOI:** 10.1001/jamanetworkopen.2022.13521

**Published:** 2022-05-23

**Authors:** Maria C. Cusimano, Nancy N. Baxter, Rinku Sutradhar, Eric McArthur, Joel G. Ray, Amit X. Garg, Simone Vigod, Andrea N. Simpson

**Affiliations:** 1 Department of Obstetrics & Gynaecology, University of Toronto, Toronto, Ontario, Canada; 2 Melbourne School of Population & Global Health, University of Melbourne, Melbourne, Victoria, Australia; 3 ICES (formerly the Institute for Clinical Evaluative Sciences), Toronto, Ontario, Canada; 4 Department of Medicine, St Michael’s Hospital/Unity Health Toronto, Toronto, Ontario, Canada; 5 Department of Medicine, Western University, London, Ontario, Canada; 6 Department of Psychiatry, Women’s College Hospital, Toronto, Ontario, Canada; 7 Department of Psychiatry, Temerty Faculty of Medicine, University of Toronto, Toronto, Ontario, Canada; 8 Department of Obstetrics & Gynaecology, St Michael’s Hospital/Unity Health Toronto, Toronto, Ontario, Canada

## Abstract

**Importance:**

Physicians may be at risk of pregnancy complications due to prolonged work hours, overnight shifts, occupational hazards, and older maternal age at first birth compared with nonphysicians. Observational studies of physicians, including comparisons across physician specialties, are needed.

**Objective:**

To compare adverse maternal and perinatal outcomes between pregnant physicians and nonphysicians and between physicians of different specialties.

**Design, Setting, and Participants:**

A population-based retrospective cohort study was conducted in Ontario, Canada. Participants included physicians and nonphysician comparators residing in high-income areas who experienced a birth at 20 or more weeks’ gestation from April 1, 2002, to November 26, 2018. Data analysis was performed from December 2020 to March 2022.

**Exposures:**

Physician occupation and physician specialty.

**Main Outcomes and Measures:**

Severe maternal morbidity (in pregnancy and up to 42 days’ post partum) and severe neonatal morbidity (up to hospital discharge among live-born infants) were the primary outcomes. Logistic regression under a generalized estimating equations approach was used to compare outcomes between physicians and nonphysicians, accounting for potentially more than 1 pregnancy per woman. Odds ratios were adjusted (aOR) for maternal age, parity, previous preterm birth, calendar year, immigration status, comorbidities, multiple gestation, and mode of delivery.

**Results:**

A total of 10 489 births occurred among 6161 licensed physicians, and 298 683 births occurred among 211 191 nonphysician counterparts. Physicians were older (median [IQR] age, 34 [31-36] vs 32 [29-35] years) and more likely to be nulliparous (5049 [48.1%] vs 128 961 [43.2%]) compared with nonphysicians. Severe maternal morbidity was more likely to occur among physicians than nonphysicians (unadjusted OR, 1.21; 95% CI, 1.04-1.41) but not after adjusting for study covariates (aOR, 1.13; 95% CI, 0.97-1.32). Severe neonatal morbidity was less likely to occur among infants of physicians than infants of nonphysicians (aOR, 0.79; 95% CI, 0.72-0.87). Compared with family physicians, neither nonsurgical specialists (aOR, 1.12; 95% CI, 0.82-1.53) nor surgical specialists (aOR, 1.43; 95% CI, 0.74-2.76) were at increased risk of severe maternal morbidity. Similar findings were observed for severe neonatal morbidity (nonsurgical specialists: aOR, 0.98; 95% CI, 0.80-1.19; surgical specialists: aOR, 1.08; 95% CI, 0.68-1.71).

**Conclusions and Relevance:**

The findings of this study suggest that female physicians may be at slightly higher risk of severe maternal morbidity. This association appeared to be mediated by their tendency to delay childbearing compared with nonphysicians. Newborns of physicians appear to experience less morbidity. Such differences were not observed between physician specialty groups.

## Key Points

**Question:**

Are physicians at an increased risk of adverse pregnancy outcomes compared with nonphysicians?

**Findings:**

In this cohort study of 10 489 births in 6161 physicians, there was an increased risk of severe maternal morbidity among physicians compared with high-income nonphysicians; however, this association was not found after adjustment for maternal age and other clinical factors. No difference in adverse outcome was observed when comparing physician specialty groups.

**Meaning:**

The findings of this study suggest that observed associations between physician occupation and pregnancy complications are likely mediated by advanced maternal age rather than the nature of the occupation.

## Teaser

This cohort study examines the rates of severe maternal and neonatal morbidity in physicians compared with nonphysicians in Ontario, Canada.

## Introduction

Physicians may be at increased risk of adverse pregnancy outcomes due to the nature of their work. Physicians often delay childbearing until after postgraduate training when they are at an advanced maternal age,^1,2^ work prolonged hours or overnight shifts,^3,4^ and may be exposed to infectious pathogens and radiation.^5,6^ All of these factors may be associated with complications during pregnancy.^7,8,9,10^

Despite the biologic plausibility of an association between physician occupation and adverse pregnancy outcomes, study findings vary widely: some report that physicians are at increased risk of certain adverse outcomes, such as hypertensive disorders and threatened preterm labor,^3,11,12,13,14,15,16^ but others documented no such association.^17,18^ Two Finnish cohort studies found that physicians were not at increased risk of preterm birth, stillbirth, or having infants who were small for gestational age (SGA) compared with white collar workers.^17,18^ Other studies are survey based and thus susceptible to sampling and misclassification bias due to participant self-report.^3,11,12,13,14,16^

Additional epidemiologic studies using high-quality data sources are needed to clarify the association between physician occupation and adverse pregnancy outcomes. Such research would provide important data on physician health in pregnancy and may help inform both preconception and prenatal counseling and care. We therefore examined the odds of maternal and neonatal morbidity in physicians compared with nonphysicians using a unique linkage of physician registration data with population-based administrative data in Ontario, Canada.^19^

## Methods

### Study Population and Design

We performed a population-based retrospective cohort study using linked health administrative databases held at ICES, a nonprofit research institute authorized to collect and analyze health care and demographic data on all residents of Ontario, Canada, without consent for health system evaluation and improvement. All residents of Ontario have access to hospital care and physician services through the universal, publicly funded Ontario Health Insurance Plan; therefore, these data are comprehensive. The study protocol was published^19^ and approved by the research ethics board at St Michael’s Hospital, Toronto, Ontario. Data were deidentified. This study followed the Strengthening the Reporting of Observational Studies in Epidemiology (STROBE) reporting guideline for cohort studies.

We included all women aged 20 to 50 years in Ontario, Canada, who had a live birth or stillbirth at 20 or more weeks’ gestation from April 1, 2002, to November 26, 2018. We excluded women who were not eligible for the Ontario Health Insurance Plan or women whose Ontario Health Insurance Plan coverage was interrupted during pregnancy to allow complete ascertainment of obstetric outcomes, and we excluded women residing in rural areas because neighborhood-level income quintile (a socioeconomic index routinely used in Ontario owing to a lack of individual-level data) is inaccurate in rural areas.^20,21^ To create an appropriate comparison group and limit confounding due to socioeconomic status, we only included nonphysicians living in the highest-income quintile level (Figure).^20,21^

**Figure.**
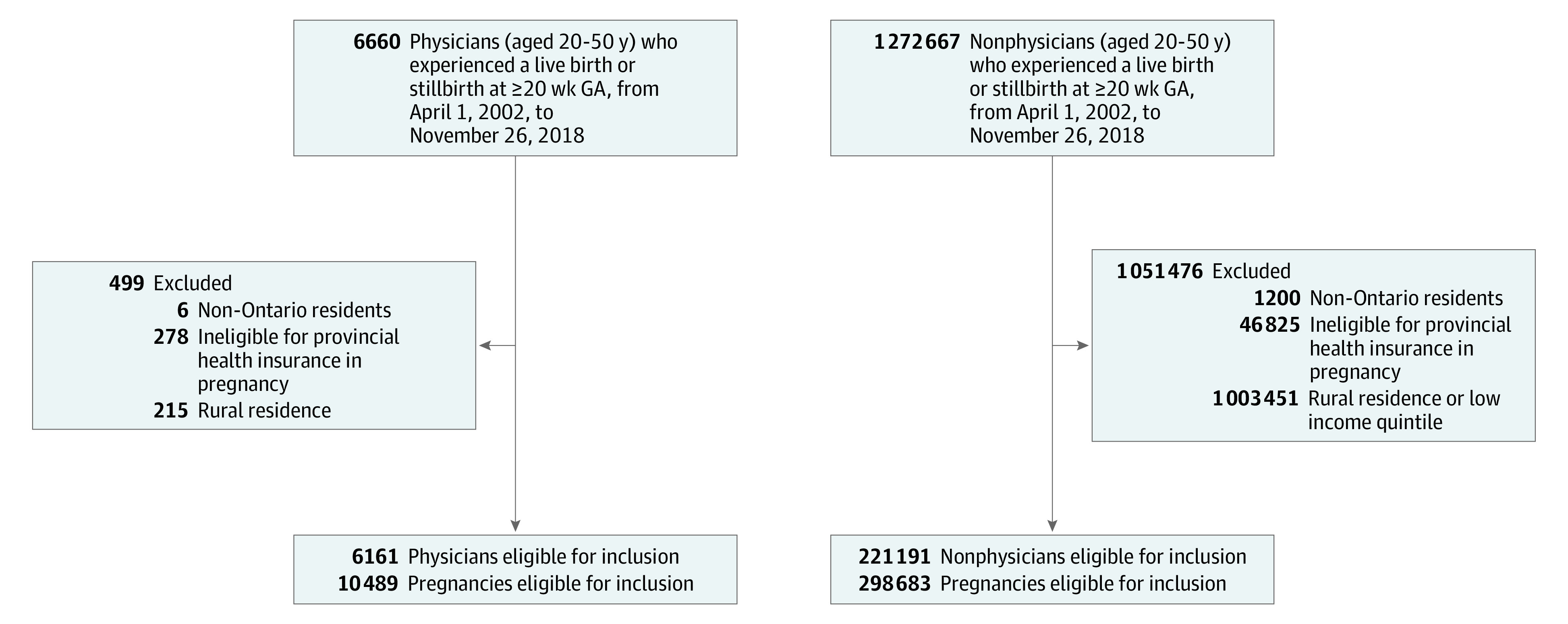
Flowchart of Included Patients GA indicates gestational age.

The study design and analyses permitted more than 1 birth to a woman during the study period. We used the ICES-derived MOMBABY database, which links the inpatient records of mothers and their newborns in Ontario, to identify all eligible pregnancies (Figure). Because more than 97% of Ontario births occur in hospitals, use of the MOMBABY database ensured capture of a representative sample of both physician and nonphysician pregnancies.^22^

### Exposure Assessment

The primary exposure was physician occupation, defined as a record of licensure as a postgraduate trainee or independent practitioner with the College of Physicians & Surgeons of Ontario at the time of the index pregnancy. The College of Physicians & Surgeons of Ontario is the sole regulatory body that grants medical licenses in the province of Ontario; therefore, assessment of physician occupation was comprehensive. We obtained this information by linking a unique data set of physicians registered with the College of Physicians & Surgeons of Ontario to existing administrative databases held at ICES.^19^

The secondary exposure was physician specialty. Nonsurgical specialists and surgical specialists were compared with family physicians. Owing to limited sample sizes, we were unable to examine individual specialties.

### Outcome Assessment

The primary maternal and perinatal outcomes were severe maternal morbidity (SMM) and severe neonatal morbidity (SNM); both outcomes were selected to capture clinically significant complications in mothers and newborns. Severe maternal morbidity is a composite end point of maternal death, as well as diagnostic (eg, eclampsia) and procedural (eg, peripartum hysterectomy) indicators that occur in pregnancy or up to 42 days post partum^23,24,25^ (eTable 1 in the Supplement). Severe neonatal morbidity is a composite end point of neonatal death, as well as diagnostic (eg, hypoxic ischemic encephalopathy) and procedural (eg, resuscitation) indicators that arise from the time of live birth to discharge home from the hospital. We used a Canadian approach to SNM^24^ like those validated in the UK and Australia^25,26^ (eTable 2 in the Supplement).

Secondary outcomes included maternal hypertensive disorders (defined as gestational hypertension, preeclampsia, or eclampsia in the index pregnancy), preterm birth less than 37 weeks’ GA, severe SGA (defined as birth weight less than the fifth percentile for sex and GA), and stillbirth at more than 20 weeks’ GA. These complications were selected owing to their clinical frequency and importance to patients and ascertained from the Canadian Institute of Health Information Discharge Abstract Database (CIHI-DAD) using standard diagnostic codes (eTable 3 in the Supplement). We selected an SGA threshold of less than the 5th percentile for our primary analyses in an attempt to capture true pathologic findings rather than constitutionally small infants in administrative data sets.

### Cohort Description and Covariates

Demographic characteristics were ascertained at the time of the index birth and included maternal age (continuous), immigration status (recent immigrant, Canadian born),^27^ calendar year (continuous), comorbidity index (0-5, 6-9, or ≥10), and specific comorbidities (ie, prepregnancy hypertension, diabetes). Comorbidities were captured in the 2 years before the index birth and categorized into aggregated diagnosis groups (ADGs) on the basis of clinical similarity, degree of disability, chronicity, and need for specialty care using the Johns Hopkins ACG System, version 10; this system ascertains health care use as a proxy for comorbidity.^28^ Specific comorbidities were ascertained from validated registries of affected Ontarians (eTable 3 in the Supplement).^29,30,31^ Comprehensive data on race or ethnicity were not available.

Pregnancy characteristics were also ascertained at the time of the index birth and included parity and history of preterm birth (categorized as no previous birth, 1 previous term birth, 1 previous preterm birth, ≥2 previous births with all term, or ≥2 previous births with at least 1 preterm), multiple gestation (singleton, twins, or higher order), and mode of delivery (spontaneous vaginal, operative vaginal, or cesarean delivery). Parity and multiple status were ascertained from standard variables in the CIHI-DAD and MOMBABY databases; mode of delivery was ascertained from procedure codes in the CIHI-DAD, which holds records of inpatient procedures, and the Ontario Health Insurance Plan Database, which holds records of physician billings (eTable 3 in the Supplement).

### Statistical Analyses

Data analysis was performed from December 2020 to March 2022. Data sets were linked using unique encoded identifiers and analyzed at ICES. We quantified differences in the means and proportions of baseline characteristics between groups using standardized differences, which are not directly dependent on population size and thus are useful when assessing balance in large population-based matched cohorts.^32^

We used logistic regression under a generalized estimating equations approach to account for potential clustering owing to multiple pregnancies per woman. Odds ratios (ORs) were generated for each outcome. Models were constructed in 3 stages for each physician vs nonphysician comparison: (1) unadjusted; (2) multivariable-adjusted for calendar year, immigration status, comorbidity index, parity, previous preterm birth, singleton vs multiple gestation, and mode of delivery, without maternal age; and (3) adding maternal age to the multivariable model. We used this sequential approach to understand the association between maternal age and any of the factors evaluated because physicians are known to delay childbearing compared with nonphysicians.^1^ Covariates for multivariable models were chosen a priori based on the literature. We present unadjusted ORs and adjusted ORs (aORs) with 95% CIs. Unadjusted absolute risk differences were obtained for SMM and SNM specifically using binomial models with an identity link.^33^ In the evaluation of SMM and SNM across physician specialties (nonsurgical specialists and surgical specialists compared with family physicians), similar models were constructed and ORs were adjusted for the included covariates.

To ensure that our findings for SMM and SNM were robust, we performed sensitivity analyses modeling these outcomes as count variables rather than binary variables with Poisson regression under a generalized estimating equations approach; we present relative rate ratios with 95% CIs for these analyses. Overall comorbidities are often associated with adverse maternal outcomes; therefore, we explored an interaction between SMM and overall comorbidities. We used a threshold of less than the 5th percentile for severe SGA to attempt to capture true pathologic findings. To further explore whether differences in neonatal birth weight might be pathologic or constitutional, we repeated SGA analyses using a threshold of less than the 10th percentile for sex and GA, consistent with standard definitions,^34^ and evaluated the odds of SNM and preterm birth only among women with live-born infants at less than the 10th percentile for sex and GA. Because trainees in Ontario have restrictions on overnight work after 27 weeks’ gestation, we performed a sensitivity analysis excluding trainees to increase generalizability to other settings.

All statistical tests were 2-sided, with differences at *P* < .05 considered statistically significant and standardized differences greater than or equal to 0.1 considered meaningful. Complete case analyses were performed because data were infrequently missing (4.4% for specialty; <0.5% for previous births). Analyses were performed in SAS, version 9.4 (SAS Institute Inc).

## Results

### Study Population

A total of 6161 physicians experiencing 10 489 pregnancies and 221 191 nonphysicians experiencing 298 638 pregnancies met the inclusion criteria (Figure). Physicians were older (median [IQR] age, 34 [31-36] vs 32 [29-35] years), more likely to be nulliparous (median [IQR], 5049 [48.1%] vs 128 961 [43.2%]), and more likely to have few comorbidities (6120 [58.3%] vs 119 257 [40.1%]) than nonphysicians at the time of their pregnancies; physicians were also more likely to experience pregnancy from 2010 to 2018 (6781 [64.6%] vs 162 925 [54.6%]) (Table 1).

**Table 1.** Characteristics of Physician and Nonphysician Pregnancies

Characteristic	No. (%)		Standardized difference
	Physicians (n = 10 489)	Nonphysicians (n = 298 638)	
Age at delivery, median (IQR), y	34 (31-36)	32 (29-35)	0.39
Year of delivery			
2002-2009	3708 (35.4)	135 713 (45.4)	0.20
2010-2018	6781 (64.6)	162 925 (54.6)	0.20
Immigration status			
Canadian born	8606 (82.0)	247 530 (82.9)	0.02
Recent immigrant	1883 (18.0)	51 108 (17.1)	0.02
Comorbidities (ADGs)			
0-5	6120 (58.3)	119 874 (40.1)	0.37
6-9	3717 (35.4)	136 835 (45.8)	0.21
≥10	652 (6.2)	41 929 (14.1)	0.26
Prepregnancy hypertension			
Yes	198 (1.9)	7302 (2.4)	0.03
No	10 291 (98.1)	291 336 (97.6)	
Prepregnancy diabetes			
Yes	83 (0.8)	3916 (1.3)	0.05
No	10 406 (99.2)	294 722 (98.7)	
Previous births^a^^,^^b^			
0	5054 (48.2)	129 182 (43.2)	0.10
1 Previous term birth	3611 (34.4)	110 237 (36.9)	0.05
1 Previous preterm birth	227 (2.2)	6477 (2.2)	0.00
≥2 Previous births, all term	1446 (13.8)	47 942 (16.1)	0.60
≥2 Previous births, ≥1 preterm	151 (1.4)	4800 (1.6)	0.02
Multiple status			
Singleton	9903 (94.4)	285 795 (95.7)	0.06
Twins or higher order	586 (5.6)	12 843 (4.3)	
Mode of delivery^c^			
Spontaneous vaginal	6089 (58.0)	178 037 (59.6)	0.03
Operative vaginal	1236 (11.8)	32 102 (10.7)	0.03
Cesarean delivery	3164 (30.2)	88 499 (29.6)	0.01

Abbreviation: ADG, aggregated diagnosis group.

^a^ Data on previous births was missing for less than 6 physicians and 221 nonphysicians.

^b^ Per ICES deidentification policy, a row with the exact number of patients missing data on previous births cannot be reported because the value was less than 6.

^c^ If mode of delivery was missing, it was assumed to be a spontaneous vaginal delivery in accordance with previous coding standards.

### Severe Maternal and Neonatal Morbidity

Severe maternal morbidity occurred in 2.1% of physician pregnancies (216 of 10 489) and 1.7% of nonphysician pregnancies (5137 of 298 638). Physicians were therefore at increased odds of SMM compared with nonphysicians (unadjusted OR, 1.21; 95% CI, 1.04-1.41; *P* = .01; unadjusted absolute difference, 3.6%; 95% CI, 0.7%-2.1%) (Table 2). The difference persisted after multivariable adjustment (aOR, 1.20; 95% CI, 1.03-1.39; *P* = .02) but was no longer statistically significant after adjusting for maternal age (aOR, 1.13; 95% CI, 0.97-1.32; *P* = .10) (eTable 4 in the Supplement). Associations were similar when SMM was treated as a count (eTable 5 in the Supplement).

**Table 2.** Adverse Maternal and Perinatal Outcomes in Physicians vs Nonphysicians

Outcome	Exposure	No. of outcome events (%)	Odds ratio (95% CI)		
			Unadjusted	Adjusted, without maternal age^a^	Adjusted, with maternal age^a^
Severe maternal morbidity	Nonphysicians (n = 298 638)	5137 (1.7)	1 [Reference]	1 [Reference]	1 [Reference]
	Physicians (n = 10 489)	216 (2.1)	1.21 (1.04-1.41)	1.20 (1.03-1.39)	1.13 (0.97-1.32)
Severe neonatal morbidity	Nonphysicians (n = 298 026)	18 406 (6.2)	1 [Reference]	1 [Reference]	1 [Reference]
	Physicians (n = 10 469)	554 (5.3)	0.86 (0.78-0.94)	0.80 (0.73-0.88)	0.79 (0.72-0.87)
Hypertensive disorders	Nonphysicians (n = 298 638)	22 046 (7.4)	1 [Reference]	1 [Reference]	1 [Reference]
	Physicians (n = 10 489)	687 (6.6)	0.91 (0.83-0.99)	0.89 (0.82-0.98)	0.86 (0.79-0.94)
Preterm birth	Nonphysicians (n = 298 638)	23 719 (7.9)	1 [Reference]	1 [Reference]	1 [Reference]
	Physicians (n = 10 489)	903 (8.6)	1.11 (1.03-1.21)	1.05 (0.96-1.15)	1.03 (0.94-1.12)
Severe SGA (<5th percentile)	Nonphysicians (n = 298 638)	11 851 (4.0)	1 [Reference]	1 [Reference]	1 [Reference]
	Physicians (n = 10 489)	518 (4.9)	1.26 (1.14-1.39)	1.18 (1.07-1.30)	1.17 (1.06-1.29)
Stillbirth	Nonphysicians (n = 298 638)	612 (0.2)	1 [Reference]	1 [Reference]	1 [Reference]
	Physicians (n = 10 489)	20 (0.2)	0.98 (0.63-1.53)	0.86 (0.55-1.35)	0.81 (0.51-1.27)

Abbreviation: SGA, small for gestational age.

^a^ Adjusted for maternal age, calendar year, immigration status, comorbidity index, parity, history of preterm birth, multiple gestation, and mode of delivery.

Severe neonatal morbidity occurred in 5.3% of physician live births (554 of 10 469) and 6.2% of nonphysician live births (18 406 of 298 026). Physicians were therefore at decreased odds of SNM compared with nonphysicians (unadjusted OR, 0.86; 95% CI, 0.78-0.94; *P* = .001; unadjusted absolute difference, −0.8%; 95% CI, −1.3% to −0.4%) (Table 2). This difference persisted after multivariable adjustment (aOR, 0.80; 95% CI, 0.73-0.88; *P* < .001) and after adjustment for maternal age (aOR, 0.79; 95% CI, 0.72-0.87; *P* < .001) (eTable 4 in the Supplement). Associations were similar when SNM was treated as a count (eTable 5 in the Supplement). The diagnostic and procedural indicators arising most commonly for both SMM and SNM are outlined in eTable 6 in the Supplement.

### Secondary Outcomes

Physicians were at increased odds of preterm birth compared with nonphysicians in unadjusted analyses (8.6% vs 7.9%; OR, 1.11; 95% CI, 1.03-1.21; *P* = .01), but this association was not found in multivariable-adjusted (without age) (aOR, 1.05; 0.96-1.15; *P* = .29) and fully adjusted (with age) analyses (aOR, 1.03; 95% CI, 0.94-1.12; *P* = .58) (Table 2).

Physicians were at decreased odds of hypertensive disorders of pregnancy (6.6% vs 7.4%; unadjusted OR, 0.91; 95% CI, 0.83-0.99; *P* = .03) but at increased odds of delivering an SGA infant in the less than 5th percentile (4.9% vs 4.0%; unadjusted OR, 1.26; 95% CI, 1.14-1.39; *P* < .001) compared with nonphysicians. These findings were also directionally similar and statistically significant in multivariable analyses, without and with maternal age (Table 2). Stillbirth was a rare event (0.2% overall), and the odds were not significantly different between physicians and nonphysicians (fully adjusted OR, 0.81; 95% CI, 0.51-1.27; *P* = .36).

### Sensitivity and Specialty-Specific Analyses

We explored a 2-way interaction between SMM and overall comorbidities (ADGs: 0-5, 6-9, or ≥10). There was no significant interaction between comorbidities and physician occupation on SMM. Physicians were also at increased odds of delivering an SGA infant less than the 10th percentile (10.4% vs 8.3%; unadjusted OR, 1.28; 95% CI, 1.19-1.38; *P* < .001). After restricting the study population to women delivering an infant at less than the 10th percentile, physicians were at similar odds of preterm birth (aOR, 0.99; 95% CI, 0.79-1.25; *P* = .93) and decreased odds of SNM (aOR, 0.72; 95% CI, 0.56-0.93; *P* = .01) compared with nonphysicians. Thus, although physicians were at increased odds of delivering an SGA infant overall, these infants were not more likely to be born preterm and were less likely to experience severe morbidity.

We removed trainees from our multivariable adjusted models, and our results were similar for SMM (aOR, 0.95; 95% CI, 0.76-1.20; *P* = .67) and SNM (aOR, 0.69; 95% CI, 0.60-0.80; *P* < .001). For practicing physicians, a total of 10 022 physician pregnancies (95.5%) had data on specialty available and were included in specialty-specific analyses (eTable 7 in the Supplement). Compared with family physicians, the adjusted odds of SMM and SNM were not significantly different for surgical specialists (SMM: aOR, 1.43; 95% CI, 0.74-2.76; *P* = .28; SNM: aOR, 1.08; 95% CI, 0.68-1.71; *P* = .74) and nonsurgical specialists (SMM: aOR, 1.12; 95% CI, 0.82-1.53; *P* = .50; SNM: aOR, 0.98; 95% CI, 0.80-1.19; *P* = .83) (Table 3). A summary of directional outcomes in the present study as well as in other published and current survey and cohort studies appears in Table 4.^3,11,12,13,14,16,17,18,35,36^

**Table 3.** Adverse Maternal and Perinatal Outcomes in Nonsurgical and Surgical Specialists vs Family Physicians

Outcome	Specialty	Odds ratio (95% CI)	
		Unadjusted	Adjusted^a^
Severe maternal morbidity	Family physician (n = 5315)	1 [Reference]	1 [Reference]
	Nonsurgical specialist (n = 4163)	1.21 (0.89-1.64)	1.12 (0.82-1.53)
	Surgical specialist (n = 544)	1.59 (0.83-3.04)	1.43 (0.74-2.76)
Severe neonatal morbidity	Family physician (n = 5305)	1 [Reference]	1 [Reference]
	Nonsurgical specialist (n = 4158)	1.01 (0.83-1.22)	0.98 (0.80-1.19)
	Surgical specialist (n = 543)	1.15 (0.73-1.80)	1.08 (0.68-1.71)

^a^ Adjusted for maternal age, calendar year, immigration status, comorbidity index, parity, history of preterm birth, multiple gestation, and mode of delivery.

**Table 4.** Summary of Published and Current Evidence on Adverse Pregnancy Outcomes Among Physicians

Source	Country	Specialty	Exposed	Comparator	Response rate, %	Direction of outcome		Covariates
						Maternal	Perinatal	
**Survey studies**								
Klebanoff et al,^11^ 1990	US	All	Women residents (n = 989)	Partners of male residents (n = 1239)	86	Increased HTN disorders and preterm labor	No significant difference in SGA, or preterm birth; stillbirth	Age, parity, height, weight, ethnicity
Osborn et al,^12^ 1990	US	All	Women residents (n = 92)	Partners of male residents (n = 144)	57	No significant difference in HTN disorders; increased preterm labor	No significant difference in preterm birth or stillbirth	None
Pinhas-Hamiel et al,^13^ 1999	Israel	All	Women physicians (n = 207)	General population (NR)	52	No significant difference in HTN disorders	Increased preterm birth and stillbirth	None
Gabbe et al,^3^ 2003	US	Obstetrics/gynecology	Women residents (n = 302)	Partners of male residents (n = 274)	96	Increased HTN disorders and preterm labor	Increased SGA; no significant difference in stillbirth	None
Lerner et al,^35^2009	US	Urology	Women surgeons (n = 243)	General population (NR)	69	Increased pregnancy complications composite	NR	None
Hamilton and Tulandi,^36^2012	US	Surgery	Women surgeons (n = 1021)	General population (NR)	NR	NR	Increased preterm birth	NR
Behbehani et al,^14^ 2015	Canada	Family medicine	Women residents (n = 238)	General population (n = 3767)	NR	Increased HTN disorders; no significant difference in preterm labor	Increased SGA	None
Rangel et al,^16^ 2021	US	Surgery	Women surgeons (n = 692)	Partners of male surgeons (n = 158)	NR	Increased pregnancy complications composite	NR	Age, race and ethnicity, hours worked per week, multiple gestation, IVF use
**Cohort studies**								
Heinonen and Saarikoski,^17^ 2002	Finland	All	Women physicians (n = 331)	General population (n = 21 997)	NA	Decreased HTN disorders	No significant difference in SGA, preterm birth, and stillbirth	Age, marital status, smoking, obesity, infertility treatment, prior terminations
Quansah et al,^18^ 2009	Finland	All	Women physician pregnancies (n = 7642)	Upper white collar worker pregnancies (n = 124 606)	NA	NR	No significant difference in SGA, preterm birth, and stillbirth	Age, parity, smoking, marital status
Current	Canada	All	Women physician pregnancies (n = 10 489)	High-income nonphysician pregnancies (n = 298 638)	NA	No significant difference in SMM; decreased HTN disorders	Decreased SNM, no significant difference in preterm birth, and increased SGA	Age at delivery, year of delivery, immigration status, comorbidities, prepregnancy hypertension, prepregnancy diabetes, previous births, multiple status, mode of delivery

Abbreviations: HTN, hypertensive; IVF, in vitro fertilization; NA, not applicable; NR, not reported; SGA, small for gestational age birth weight; SMM, severe maternal morbidity; SNM, severe neonatal morbidity.

## Discussion

In this population-based cohort study including more than 309 000 unique pregnancies, physicians were more likely to experience SMM than high-income nonphysicians but were also older and more often nulliparous at the time of birth. After controlling for maternal age, parity, and other confounders, physicians were no more likely to experience adverse pregnancy outcomes, and their infants were significantly less likely to experience severe neonatal morbidity. Among physicians, there also were no significant differences in maternal or perinatal morbidity based on specialty.

Results of our study suggest that physicians are not inherently at increased risk of adverse pregnancy outcomes compared with nonphysicians; rather, any potential association between physician occupation and adverse outcomes may be mediated by advanced maternal age owing to delay of childbirth.^1^ This finding is consistent with other registry-based cohort studies (Table 4). Heinonen and Saarikoski^17^ compared 331 physicians, 656 teachers, and 21 997 general population controls; after multivariable adjustment, there were no significant differences in the rates of preterm birth, fetal distress at delivery, or neonatal intensive care use between groups. Quansah et al^18^ compared 7642 physicians with 124 606 upper white collar workers and similarly found no significant differences in the rates of preterm birth or perinatal death. Numerous survey-based studies have contrastingly suggested that women physicians may be at increased risk of certain adverse pregnancy outcomes compared with the general population or partners of male physicians^3,11,12,13,14,16^; however, the associations observed in these studies are inconsistent, often unadjusted for important factors, and prone to sampling bias in that physicians responding to a survey may have a higher incidence of complications than nonparticipants or the general population (Table 4). Given the methods applied in our study and the consistency of published observational studies, it appears to be unlikely that physician occupation itself increases adverse pregnancy outcomes.

After controlling for age and other factors, we found that physicians were at increased risk of only 1 adverse outcome: delivering an SGA infant. Our percentile-based definitions for SGA, required as a result of our use of administrative data,^37^ could not distinguish between constitutionally small infants and infants who were growth restricted due to underlying pathologic factors.^38^ It is possible that the association observed may represent an increase in constitutionally small infants among physicians. First, sensitivity analyses restricted to women with SGA infants suggested that physicians’ infants were at a reduced risk of perinatal morbidity and no more likely to be born preterm; this finding is consistent with work by Ananth and Vintzileos^37^ suggesting that constitutionally small infants have decreased mortality and are more often delivered at term. Second, physicians had fewer comorbidities and decreased odds of hypertensive disorders; the opposite would be expected in the setting of intrauterine growth restriction.^39^ We were also unable to control for maternal obesity and ethnicity, which may have explained the association observed: Heinonen and Saarikoski^17^ reported that physicians are less likely to have obesity than the general population and were not at increased risk of SGA after adjusting for maternal obesity and other factors (OR, 0.99; 95% CI, 0.68-1.50). However, occupational factors, such as prolonged hours and rotating shifts, have been associated with SGA in other studies^10^; therefore, the association between physician occupation and SGA warrants further investigation.

Our findings highlight the risks of delaying childbirth to an advanced maternal age. Although elective oocyte cryopreservation has been increasingly proposed as a way for women physicians to overcome age-related infertility, it is important to recognize that this strategy will not prevent severe maternal morbidity mediated by delayed childbirth and advanced maternal age.^40^

### Strengths and Limitations

To our knowledge, this cohort study is the largest to date to evaluate adverse pregnancy outcomes in physicians. In contrast to surveys, we studied a representative cohort of pregnancies among postgraduate trainees and independent physicians in multiple specialties and ascertained adverse pregnancy outcomes with little risk of misclassification. We used validated outcome measures that reflect substantial morbidity, and our sequential approach to statistical analysis clarifies mechanistic factors in the exposure-outcome association.

The study has limitations. First, we lacked data on occupation, educational attainment, and individual-level income for nonphysicians. To ensure as fair a comparison as possible and mitigate the potential for confounding due to socioeconomic status, we compared physicians with nonphysicians living only in high-income neighborhoods. Second, we were unable to control for other factors, such as maternal ethnicity, body habitus, smoking, and substance use; however, many of these factors are likely to be further protective for physicians. Third, we lacked comprehensive data on use of assisted reproductive technology, a known risk factor for adverse pregnancy outcomes,^41^ because such services are primarily privately funded in Ontario. However, assisted reproductive technology use is likely collinear with covariates for maternal age and comorbidity, and if physicians use assisted reproductive technology more than nonphysicians, resulting aORs should be even lower than those we noted. Fourth, we could not examine spontaneous abortion and early pregnancy complications because they are not accurately captured in administrative databases. Fifth, this study was performed in a single Canadian province and may not be generalizable to jurisdictions with differing clinical expectations and leave options for physician parents. In Ontario, residents are exempt from on-call shifts after 27 weeks’ GA and entitled up to 52 weeks of partially paid pregnancy/parental leave^42^; however, independent practitioners may continue on-call shifts late into pregnancy and have access to a 17-week pregnancy/parental leave benefit.^43^ To address this limitation, we performed a sensitivity analysis evaluating only practicing physicians, and our results did not change.

## Conclusions

Results of this cohort study suggest that physician occupation may be associated with pregnancy complications but that this association is likely mediated by delayed childbearing and advanced maternal age at first birth rather than the nature of the occupation. Additional observational studies are needed to examine further work characteristics, such as intensity of overnight shifts and work hours, on specific adverse pregnancy outcomes.

## Notes

Supplement. **eTable 1.** *International Classification of Diseases* (*ICD*) and Canadian Classification of Intervention (CCI) Codes to Identify Severe Maternal Morbidity (SMM) **eTable 2.** *International Classification of Diseases* (*ICD*) and Canadian Classification of Intervention (CCI) Codes to Identify Severe Neonatal Morbidity (SNM) **eTable 3.** Codes to Identify Specific Covariates and Maternal/Perinatal Complications **eTable 4.** Multivariable Analyses for Primary Maternal and Perinatal Outcomes **eTable 5.** Severe Maternal Morbidity and Severe Neonatal Morbidity, Modelled as Count Rather Than Binary Variables **eTable 6.** Most Frequent Diagnostic and Procedural Indicators for Primary Outcomes Among Physicians and Nonphysicians Experiencing Severe Maternal Morbidity and Severe Neonatal Morbidity, Respectively **eTable 7.** Baseline Characteristics of Pregnancies in Physicians by Specialty
