# Supplementary material for: Evaluation of Adverse Pregnancy Outcomes in Physicians Compared With Nonphysicians
Source: JAMA Netw Open. 2022 May 23;5(5):e2213521. doi: 10.1001/jamanetworkopen.2022.13521 (PMC9127555; doi:10.1001/jamanetworkopen.2022.13521)
Supplement: Supplement. — eTable 1. International Classification of Diseases (ICD) and Canadian Classification of Intervention (CCI) Codes to Identify Severe Maternal Morbidity (SMM) eTable 2. International Classification of Diseases (ICD) and Canadian Classification of Intervention (CCI) Codes to Identify Severe Neonatal Morbidity (SNM) eTable 3. Codes to Identify Specific Covariates and Maternal/Perinatal Complications eTable 4. Multivariable Analyses for Primary Maternal and Perinatal Outcomes eTable 5. Severe Maternal Morbidity and Severe Neonatal Morbidity, Modelled as Count Rather Than Binary Variables eTable 6. Most Frequent Diagnostic and Procedural Indicators for Primary Outcomes Among Physicians and Nonphysicians Experiencing Severe Maternal Morbidity and Severe Neonatal Morbidity, Respectively eTable 7. Baseline Characteristics of Pregnancies in Physicians by Specialty [file jamanetwopen-e2213521-s001.pdf]

Supplementary Online Content

Cusimano MC, Baxter NN, Sutradhar R, et al. Evaluation of adverse pregnancy outcomes in physicians compared with nonphysicians. *JAMA Netw Open*. 2022;5(5):e2213521. doi:10.1001/jamanetworkopen.2022.13521

eTable 1. *International Classification of Diseases* (*ICD*) and Canadian Classification of Intervention (CCI) Codes to Identify Severe Maternal Morbidity (SMM)

eTable 2. *International Classification of Diseases* (*ICD*) and Canadian Classification of Intervention (CCI) Codes to Identify Severe Neonatal Morbidity (SNM)

eTable 3. Codes to Identify Specific Covariates and Maternal/Perinatal Complications

eTable 4. Multivariable Analyses for Primary Maternal and Perinatal Outcomes

eTable 5. Severe Maternal Morbidity and Severe Neonatal Morbidity, Modelled as Count Rather Than Binary Variables

eTable 6. Most Frequent Diagnostic and Procedural Indicators for Primary Outcomes Among Physicians and Nonphysicians Experiencing Severe Maternal Morbidity and Severe Neonatal Morbidity, Respectively

eTable 7. Baseline Characteristics of Pregnancies in Physicians by Specialty

This supplementary material has been provided by the authors to give readers additional information about their work.

© 2022 Cusimano MC et al. *JAMA Network Open.*

eTable 1. *International Classification of Diseases* (*ICD*) and Canadian Classification of Intervention (CCI) codes to identify severe maternal morbidity (SMM).

SMM Indicator	ICD or CCI Codes*
Severe preeclampsia and HELLP syndrome	O14.1 or O14.2
Eclampsia	O15
Cerebral venous thrombosis in pregnancy, or in the puerperium	O22.5 or O87.3
Acute fatty liver with red blood cell (RBC) transfusion or plasma transfusion	O26.6 + (CIHI BTREDBC = 1 or CIHI BTPLASMA = 1)
Pulmonary, cardiac, and CNS complications of anaesthesia during pregnancy, the puerperium, or labour and delivery	O29.0, O29.1, O29.2, O89.0, O89.1, O89.2, O74.0, O74.1, O74.2 or O74.3
Placenta previa with hemorrhage with RBC transfusion	O44.1 + CIHI BTREDBC = 1
Placental abruption with coagulation defect	O45.0
Antepartum hemorrhage with coagulation defect	O46.0
Intrapartum hemorrhage with coagulation defect	O67.0
Intrapartum hemorrhage with RBC transfusion	O67 + CIHI BTREDBC = 1
Rupture of the uterus with RBC transfusion, procedures to the uterus or hysterectomy	(O71.0 or O71.1) + any of the following:<br>• CIHI BTREDBC = 1, or<br>• (1.RM.13, 1.KT.51, 5.PC.91.LA or 5.PC.91.HV) + CIHI BTREDBC = 1, or<br>• (5.MD.60.RC, 5.MD.60.RD, 5.MD.60.KE, 5.MD.60.CB or 1.RM.89.LA[a]), or<br>• 1.RM.87.LA-GX<br><br>[a]**NOTE**: **1.RM.89.LA** is included only if codes 1.PL.74, 1.RS.74 or 1.RS.80 are NOT also present
Postpartum hemorrhage with RBC transfusion, procedures to the uterus or hysterectomy	O72 + any of the following:<br>• BTREDBC = 1, or<br>• (1.RM.13, 1.KT.51, 5.PC.91.LA or 5.PC.91.HV) + BTREDBC = 1, or<br>• (5.MD.60.RC, 5.MD.60.RD, 5.MD.60.KE, 5.MD.60.CB or 1.RM.89.LA[b]), or<br>• 1.RM.87.LA-GX<br><br>[b]**NOTE**: **1.RM.89.LA** is included only if codes 1.PL.74, 1.RS.74 or 1.RS.80 are NOT also present
Cardiac conditions	O74.2, O89.1, O90.3, I21, I22, I42, I43, I46, I49.0, I50, J81, 1.HZ.09 or 1.HZ.30
Obstetric shock	O75.1, R57, T80.5 or T88.6
Septicemia during labour	O75.3
Complications of obstetric surgery and procedures	O75.4
Puerperal sepsis	O85
Obstetric embolism	O88
Acute renal failure	O90.4, N17, N19 or N99.0
Death, obstetric, cause unspecified	O95
Death, obstetric, after 42 days but 1 year after delivery	O96
Death from sequelae of direct obstetric causes	O97
Disseminated intravascular coagulation	D65
Sickle cell anemia with crisis	D57.0
Acute psychosis	F53.1 or F23

© 2022 Cusimano MC et al. *JAMA Network Open.*

Status epilepticus	G41
Cerebral edema or coma	G93.6 or R40.2
Cerebrovascular diseases: subarachnoid and intracranial hemorrhage, cerebral infarction, stroke	I60, I61, I62, I63 or I64
Status asthmaticus	J45.01, J45.11, J45.81 or J45.91
Adult respiratory distress syndrome	J80
Acute abdomen	K35, K37, K65, N73.3 or N73.5
Hepatic failure	K71 or K72
Sudden death, death from unspecified cause	R96, R97, R98 or R99
Assisted ventilation through endotracheal tube	1.GZ.31.CA-ND
Assisted ventilation through tracheostomy	1.GZ.31.CR-ND
Hysterectomy	5.MD.60.RC, 5.MD.60.RD, 5.MD.60.KE, 5.MD.60.CB, 1.RM.89.LA (exclude if 1.PL.74, 1.RS.74 or 1.RS.80 code also present) or 1.RM.87.LA-GX
Dialysis	1.PZ.21
Evacuation of incisional hematoma with RBC transfusion	5.PC.73.JS + CIHI BTREDBC = 1
Repair of bladder, urethra, or intestine	5.PC.80.JR, 1.NK.80 or 1.NM.80
Procedures to the uterus/pelvic vessels with RBC transfusion	(1.RM.13, 1.KT.51, 5.PC.91.LA or 5.PC.91.HV) + CIHI BTREDBC = 1
Surgical or manual correction of inverted uterus for vaginal births only	5.PC.91.HQ or 5.PC.91.HP, restricted to vaginal births (i.e., absence of caesarean 5.MD.60)
Reclosure of caesarean wound	(5.PC.80.JM or 5.PC.80.JH) + CIHI BTREDBC = 1
Curettage with RBC transfusion	(5.PC.91.GA, 5.PC.91.GC or 5.PC.91.GD) + CIHI BTREDBC = 1
Maternal ICU admission	SCU in (‘10’,’20’,’25’,’30’,’35’,’40’,’45’,’60’,’80’)

Notes on Selected Diagnostic and Procedure Codes:

(1) Coding specific to severe preeclampsia and HELLP (O14.1 and O14.2) began in 2012.

(2) Codes under acute fatty liver (O26.6) were expanded in ICD-10-CA version 2009 to: (a) Add codes for the sixth digits of 2 (“Delivered, with mention of postpartum complication”) and 4 (“Postpartum condition or complication”), which would have previously been captured at O90.802 and O90.804 for “Other complications of the puerperium, not elsewhere classified”, respectively; and (b) Include “Cholestasis (intrahepatic) in pregnancy” and “Obstetric cholestasis,” which would previously have been classified under O99.6 for “Diseases of the digestive system complicating pregnancy, childbirth and the puerperium” which included conditions in K80-K93 and more specifically K83.1 for “Cholestasis not elsewhere classified”.

(3) CCI code 5.PC.91.HV for “Interventions to uterus (following delivery or abortion), compression using intrauterine balloon” was introduced in CCI version 2012. This intervention would previously have been captured at 5.PC.91.HT for “Interventions to uterus (following delivery or abortion) uterine (and vaginal) packing.”

© 2022 Cusimano MC et al. *JAMA Network Open.*

eTable 2. *International Classification of Diseases* (*ICD*) and Canadian Classification of Intervention (CCI) codes to identify severe neonatal morbidity (SNM).

**SNM Indicator**	**ICD or CCI Codes**
**Diagnoses**	
Gestational age <32 weeks	(from MOMBABY)
Birth weight <1500 grams	(from MOMBABY)
Neonatal death (within 28 days of birth or before a discharge home from hospital)	(from RPDB: [DTHDATE] within 28 days of index date)
Respiratory distress syndrome	P220
Seizure	P90, R56
Intraventricular hemorrhage (grades 2,3,4)	P521, P522
Cerebral infarction	I63
Periventricular leukomalacia	P912
Birth trauma (intracranial hemorrhage, paralysis due to brachial plexus injury, skull or long bone fracture)	P100 to P103, P130, P132, P133, P140, P141
Hypoxic ischemic encephalopathy	P915, P9181, P916
Necrotising enterocolitis	P77
Broncho-pulmonary dysplasia	P271
Sepsis/septicaemia (streptococcus, staphylococcus, *E.coli*, unspecified Gram-negative)	P36 (preferred code by the Public Health Agency of Canada used)
Pneumonia	P23 (preferred code by the Public Health Agency of Canada used)
Other respiratory: primary atelectasis, respiratory failure	P280, P285
Procedures	
Resuscitation	1HZ30JN, 1HZ30JY, 1GZ30CJ, 1GZ30CJNB, 1GZ30JH
Ventilatory support (mechanical ventilation and/or CPAP)	1GZ31CAEP, 1GZ31CAND, 1GZ31CAPK, 1GZ31CBND, 1GZ31CRND, 1GZ31GPND, 1GZ31JAGX, 1GZ31JAMD, 1GZ31JANC, 1GZ31JAPK
Central venous or arterial catheter	1KV53HACH, 1KV53HAFT, 1KV53LAFT, 2IM28GP, 2LZ28GQPL, 2LZ28GRPL, 2LZ28JAPL, 1KX53HACH, 1KX53HAFT, 1KX53 LAFT, 2LZ28GQPL, 2LZ28GRPL
Pneumothorax requiring intercostal catheter	1GV52DA, 1GV52DATS, 1GV52HA, 1GV52HAHE, 1GV52HATK, 1GV52LA, 1GV52LATS, 1GV52LAXXE, 1GV54JATS, 1GV55JATS
Any intravenous fluids	1LZ35CAE6, 1LZ35HAC1, 1LZ35HAC5, 1LZ35HAC6, 1LZ35HAC7, 1LZ35HAE6, 1LZ35HAT7, 1LZ35HAT9, 1LZ35HAZ9, 1LZ35HHC1, 1LZ35HHC5, 1LZ35HHC6, 1LZ35HHC7, 1LZ35HHE0, 1LZ35HHE6, 1LZ35HHT7, 1LZ35HHT9, 1LZ35HHZ9, 1LZ35HRC5, 1LZ35HRC6, 1LZ35HRC7, 1LZ35HRT9, 1LZ35HRZ9
Transfusion of blood or blood products	1LZ19HHU1A, 1LZ19HHU1J, 1LZ19HHU2A, 1LZ19HHU2J, 1LZ19HHU3J, 1LZ19HHU4J, 1LZ19HHU5J, 1LZ19HHU6A, 1LZ19HHU6J, 1LZ19HHU9A, 1LZ19HHU9J, 1LZ19HMU1, 1LZ19HMU2, 1LZ19HMU9, 1LZ35HAC5
Any body cavity surgical procedure (substring)	1AA52, 1AA87, 1AC87, 1AE87, 1AF87, 1AG87, 1AJ87, 1AK87, 1AN52, 1AN59, 1AN87, 1AP59, 1AP72, 1AP87, 1AW59, 1AW72, 1AW87, 1AX87, 1BA72, 1BA80, 1BA87, 1BB72, 1BB80, 1BB87, 1BD72, 1BD80, 1BD87, 1BF80, 1BG72, 1BG80, 1BG87, 1BK59, 1BM72, 1BM80, 1BM87, 1BN72, 1BN80, 1BN87, 1BP72, 1BP80, 1BP87, 1BQ72, 1BQ80, 1BQ87, 1BS72, 1BS80, 1BS87, 1BT72, 1BT80, 1BT87, 1GA87, 1GA89, 1GB87, 1GB89, 1GD89, 1GE80,

	1GE87, 1GE89, 1GE91, 1GH84, 1GJ86, 1GJ87, 1GK87, 1GK89, 1GM80, 1GM86, 1GM87, 1GN92, 1GR87, 1GR89, 1GR91, 1GT78, 1GT87, 1GT89, 1GT91, 1GV87, 1GV89, 1GW87, 1GX80, 1GX86, 1GX87, 1GY70, 1GY72, 1GY86, 1HJ76, 1HJ82, 1HN87, 1HP76, 1HP78, 1HP80, 1HP82, 1HP83, 1HP87, 1HR80, 1HR84, 1HR87, 1HS80 (excl. 1HS80G), 1HS90, 1HT80 (excl. 1HT80G), 1HT89, 1HT90, 1HU80 (excl. 1HU80G), 1HU90, 1HV80 (excl. 1HV80G), 1HV90, 1HW78, 1HW79, 1HX80, 1HX87, 1HX80, 1HZ87, 1IA76, 1IA80, 1IA87, 1IB76, 1IB79, 1IB80, 1IB82, 1IB87, 1IC76, 1IC80, 1IC82, 1IC87, 1ID76, 1ID80, 1ID82, 1ID86, 1ID87, 1IF83, 1IJ76, 1IJ80, 1IM76, 1IM80, 1IM82, 1IM83, 1IM87, 1IN83, 1IN84, 1IN87, 1JE57 (excl. 1JE57G), 1JE76, 1JE80, 1JE87, 1JJ76, 1JJ80, 1JK76, 1JK80, 1JK87, 1JW51 (excl. 1JW51G), 1JW57, 1JW76, 1LA84, 1LC84, 1LD84, 1NA72, 1NA74, 1NA76, 1NA77, 1NA80, 1NA84, 1NA86, 1NA87, 1NA88, 1NA89, 1NA90, 1NA91, 1NA92, 1NE80, 1NF76, 1NF78, 1NF80, 1NF82, 1NF84, 1NF86, 1NF87 (excl. 1NF87B), 1NF89, 1NF90, 1NF91, 1NF92, 1NK76, 1NK77, 1NK80, 1NK82, 1NK84, 1NK87 (excl. 1NK87B), 1NM74, 1NM76, 1NM77, 1NM80, 1NM82, 1NM87 (excl. 1NM87B), 1NM89, 1NM91, 1NP72, 1NP73, 1NP86, 1NQ74 (excl. 1NQ74B), 1NQ80, 1NQ84, 1NQ86, 1NQ87 (excl. 1NQ87B), 1NQ89, 1NQ90, 1NT80, 1NT84, 1NT86, 1NT87, 1NV89, 1OA87, 1OB87, 1OB89, 1OD76, 1OD89, 1OE76, 1OE80, 1OE89, 1OJ76 (excl. 1OJ76B), 1OJ87, 1OJ89, 1OK87, 1OK89, 1OK91, 1OT72, 1OT87, 1OT91, 1PB87, 1PB89, 1PC80, 1PC87 (excl. 1PC87D), 1PC89, 1PC91, 1PE57 (excl. 1PE57BD), 1PE80 (excl. 1PE80D), 1PE82, 1PE87 (excl. 1PE87D), 1PE89 (excl. 1PE89D), 1PG76, 1PG77, 1PG80 (excl. 1PG80D), 1PG86, 1PG89, 1PL74 (excl. 1PL74CD), 1PL80, 1PM79, 1PM86, 1PM87 (excl. 1PM87B), 1PM89, 1PM90, 1PM91, 1PM92, 1QE53, 1QE80, 1QE82, 1QE84, 1QE87, 1QE89, 1QG89, 1QM74, 1QM80, 1QM87, 1QM89, 1QM91, 1QN82, 1QT87, 1QT91, 1RB74, 1RB80, 1RB83, 1RB87, 1RB89,1RD89, 1RF51, 1RF72, 1RF74, 1RF80, 1RF87, 1RF89, 1RM87 (excl. 1RM87B), 1RM89, 1RM91, 1RN87, 1RN89, 1RS74, 1RS80, 1RS86, 1RS87, 1RS89, 1RW87, 1RW88, 1RW91, 1RW92, 1SA74, 1SA75, 1SA80, 1SA89, 1SC74, 1SC75, 1SC80, 1SC87, 1SC89, 1SE53, 1SE89 (excl. 1SE89D), 1SF80, 1SF87, 1SF89, 1SG80, 1SG87, 1SH87, 1SM74, 1SM80, 1SM87, 1SN87, 1SN93, 1SQ53, 1SQ74, 1SQ80, 1SQ87, 1SQ91, 1SQ93, 1SW74, 1SY80, 1SY84, 1SY87, 1SZ87, 1VA53, 1VA74, 1VA75, 1VA80, 1VA87, 1VA93, 1VC74, 1VC80, 1VC87, 1VC91, 1VC93, 1VE80, 1VG53, 1VG55, 1VG72, 1VG73, 1VG74, 1VG75, 1VG80, 1VG87, 1VG93, 1VK80, 1VK87, 1VK89, 1VL80, 1VL87, 1VM80, 1VM87, 1VN80, 1VN87, 1VP74, 1VP80, 1VP87, 1VP89, 1VQ74, 1VQ79, 1VQ80, 1VQ82, 1VQ87, 1VQ91, 1VQ93, 1VS72, 1VS80, 1VX87

eTable 3. Codes to identify specific covariates and maternal/perinatal complications.

Disease/Condition	Citation	Source/Codes
**Covariates**		
Hypertension	Tu et al. Prevalence and incidence of hypertension from 1995 to 2005: a population-based study. CMAJ. 2008; 178(11): 1429-1435.<br>SN: 72% / SP: 95%	**Ontario Hypertension Database**<br>ICD-9: 401, 402, 403, 404, 405<br>ICD-10: I10, I11, I12, I13, I15<br>OHIP: 401, 402, 403, 404, 405
Diabetes	Lipscombe et al. Identifying diabetes cases from administrative data: a population-based validation study. BMC Health Serv Res. 2018;18(1):316.<br>SN: 90.0% / SP: 97.7%	**Ontario Diabetes Database**<br>ICD-9: 250<br>ICD-10: E10, E11, E13, E14<br>OHIP: 250, K030, K045, K046, Q040<br>SUBCLNAM = Insulins; oral antiglycemics
**Outcomes**		
Preterm birth<br>(23-36 weeks GA)	Ray et al. Mortality in infants affected by preterm birth and severe small-for-gestational age birth weight. Pediatrics. 2017; 140(6):e20171881.	**ICES MOMBABY**<br>23 <= B_GESTWKS_DEL <= 36<br>(if missing use M_GESTWKS_DEL)
Small-for-gestational age	Ray et al. Mortality in infants affected by preterm birth and severe small-for-gestational age birth weight. Pediatrics. 2017; 140(6):e20171881.	**ICES MOMBABY**<br>B_WEIGHT <10th percentile for B_SEX and B_GESTWKS_DEL (if missing use M_GESTWKS_DEL)
Gestational hypertension	Garg AX. Gestational hypertension and pre-eclampsia in living kidney donors. N Engl J Med. 2015; 372(2): 124-133.	**CIHI-DAD**<br>ICD9: 642.0, 642.3, 642.9<br>ICD10: O13, O16
Pre-eclampsia or eclampsia	Garg AX. Gestational hypertension and pre-eclampsia in living kidney donors. N Engl J Med. 2015; 372(2): 124-133.	**CIHI-DAD**<br>ICD9: 642.4, 642.5, 642.6, 642.7<br>ICD10: O11, O14, O15<br>**OHIP:** 642
Spontaneous vaginal delivery	KS Joseph et al. Validation of perinatal data in the Discharge Abstract Database of the Canadian Institute for Health Information. Chronic Dis Can. 2009; 29(3):96-100.	**CIHI-DAD**<br>CCP: 85.6, 85.69<br>CCI: 5.MD.50.^^, 5.MD.51.^^, 5.MD.52.^^<br>**OHIP:** P006
Operative vaginal delivery	KS Joseph et al. Validation of perinatal data in the Discharge Abstract Database of the Canadian Institute for Health Information. Chronic Dis Can. 2009; 29(3):96-100.	**CIHI-DAD**<br>CCP: 84.0-4; 84.7-9<br>CCI: 5.MD.53.^^, 5.MD.54.^^, 5.MD.55.^^<br>**OHIP:** P020
Caesarean section	KS Joseph et al. Validation of perinatal data in the Discharge Abstract Database of the Canadian Institute for Health Information. Chronic Dis Can. 2009; 29(3):96-100.	**CIHI-DAD**<br>CCP: 860, 861, 862, 868, 869<br>CCI: 5.MD.60<br>**OHIP:** P018, P041, P042

Abbreviations: Canadian Institute for Health Information Discharge Abstract Database (DAD); Gestational age (GA); International Classification of Diseases (ICD); Ontario Health Insurance Plan Database (OHIP); sensitivity (SN); specificity (SP)

© 2022 Cusimano MC et al. *JAMA Network Open.*

eTable 4. Multivariable analyses for primary maternal and perinatal outcomes.

**Covariate**	**Severe Maternal Morbidity**		**Severe Neonatal Morbidity**	
	OR (95% CI)	p-value	OR (95% CI)	p-value
Physician occupation				
Yes	1.13 (0.97-1.32)	0.10	0.79 (0.72-0.87)	<0.001
No	Ref	–	Ref	–
Age				
Per year increase	1.03 (1.02-1.04)	<0.001	1.00 (1.00-1.01)	0.24
Year of birth				
Per year increase	1.04 (1.03-1.04)	<0.001	1.03 (1.02-1.03)	<0.001
Immigration status				
Canadian-born	Ref	–	Ref	–
Recent immigrant	0.99 (0.92-1.07)	0.76	0.87 (0.84-0.91)	<0.001
Comorbidities				
0-5	Ref	–	Ref	–
6-9	1.33 (1.24-1.42)	<0.001	1.16 (1.12-1.21)	<0.001
10+	1.79 (1.65-1.94)	<0.001	1.45 (1.38-1.52)	<0.001
Parity and history of preterm birth				
No prior	Ref	–	Ref	–
1 prior term	0.61 (0.57-0.65)	<0.001	0.59 (0.57-0.61)	<0.001
1 prior preterm	0.97 (0.81-1.15)	0.72	1.58 (1.45-1.72)	<0.001
2+ prior, all term	0.64 (0.58-0.70)	<0.001	0.59 (0.56-0.63)	<0.001
2+ prior, 1+ preterm	0.90 (0.73-1.10)	0.29	1.27 (1.14-1.42)	<0.001
Multiple status				
Singleton	Ref	–	Ref	–
Twins or higher order	2.39 (2.14-2.67)	<0.001	4.76 (4.50-5.04)	<0.001
Mode of delivery				
Spontaneous vaginal	Ref	–	Ref	–
Operative vaginal	1.40 (1.26-1.56)	<0.001	1.38 (1.31-1.46)	<0.001
Caesarean section	2.73 (2.56-2.91)	<0.001	1.99 (1.92-2.06)	<0.001

© 2022 Cusimano MC et al. *JAMA Network Open.*

eTable 5. Severe maternal morbidity and severe neonatal morbidity, modelled as count rather than binary variables.

Outcome	**Unadjusted**		**Age-Adjusted**		**Fully-Adjusted***	
	RR (95% CI)	p-value	RR (95% CI)	p-value	RR (95% CI)	p-value
Severe maternal morbidity	1.16 (0.98-1.38)	0.08	1.22 (1.03-1.46)	0.025	1.15 (0.97-1.37)	0.11
Severe neonatal morbidity	0.89 (0.79-0.1.01)	0.06	0.88 (0.78-0.99)	0.036	0.87 (0.77-0.98)	0.024

*Adjusted for maternal age, calendar year, immigration status, overall comorbidity burden, parity and history of preterm birth, multiple gestation, and mode of delivery

Abbreviations: CI (confidence interval), RR (rate ratio)

© 2022 Cusimano MC et al. *JAMA Network Open.*

eTable 6. Most frequent diagnostic and procedural indicators for primary outcomes among physicians and nonphysicians experiencing severe maternal morbidity and severe neonatal morbidity, respectively

Severe Maternal Morbidity					Severe Neonatal Morbidity				
**Component**	**Non-Physicians (n=5,137)**		**Physicians (n=216)**		**Component**	**Non-Physicians (n=18,406)**		**Physicians (n=554)**	
	N	%	N	%		N	%	N	%
Postpartum hemorrhage with RBC transfusion	1,410	27.4	67	31.0	Ventilatory support	12,600	68.5	415	74.9
Maternal ICU admission	848	16.5	21	9.7	Intravenous fluids	6,064	32.9	197	35.6
Puerperal sepsis	853	16.6	52	24.1	Respiratory distress syndrome	4,293	23.3	134	24.2
Severe pre-eclampsia & HELLP syndrome	660	12.8	21	9.7	Gestational age <32 weeks	3,278	17.8	101	18.2
Peripartum hysterectomy	523	10.2	25	11.6	Birthweight <1500 grams	2,893	15.7	80	14.4
Curettage with RBC transfusion	403	7.8	20	9.3	Sepsis/ septicaemia	2,806	15.2	66	11.9
Cardiac conditions	310	6.0	8	3.7	Other respiratory conditions: Primary atelectasis, respiratory failure	2,244	12.2	59	10.6
Eclampsia	243	4.7	<6	<2.8	Central venous or arterial catheter	1,155	6.3	42	7.6
Complications of obstetric surgery and procedures	247	4.8	9	4.2	Body cavity surgical procedure	811	4.4	20	3.6
Procedures to the uterus/pelvic vessels with RBC transfusion	248	4.8	12	5.6	Neonatal death	704	3.8	21	3.8

© 2022 Cusimano MC et al. *JAMA Network Open.*

eTable 7. Baseline characteristics of pregnancies in physicians by specialty.

Characteristics	Family medicine (N=5,315)	Surgical (N=544)	Other (N=4,163)
Age (years)			
Median (IQR)	33 (31-36)	35 (32-37)	34 (32-36)
Year of birth, *N (%)*			
2002-2009	1,913 (36.0%)	197 (36.2%)	1,495 (35.9%)
2010-2018	3,402 (64.0%)	347 (63.8%)	2,668 (64.1%)
Immigration status, *N (%)*			
Canadian-born	4,411 (83.0%)	469 (86.2%)	3,410 (81.9%)
Immigrant	904 (17.0%)	75 (13.8%)	753 (18.1%)
Comorbidities (ADGs), *N (%)*			
0-5	3,049 (57.3%)	349 (64.2%)	2,469 (59.3%)
6-9	1,917 (36.1%)	168 (30.9%)	1,454 (34.9%)
10+	349 (6.6%)	27 (5.0%)	240 (5.8%)
Pre-existing hypertension, *N (%)*			
Yes	87 (1.6%)	10 (1.8%)	95 (2.3%)
Pre-existing diabetes, *N (%)*			
Yes	42 (0.8%)	2 (0.4%)	36 (0.9%)
Parity and history of preterm birth, *N (%)*			
No prior births	2,498 (47.0%)	268 (49.3%)	2,039 (49.0%)
1 prior term birth	1,858 (35.0%)	180 (33.1%)	1,450 (34.8%)
1 prior preterm birth	107 (2.0%)	10 (1.8%)	102 (2.5%)
2+ prior births, all term	779 (14.7%)	75 (13.8%)	518 (12.4%)
2+ prior births, 1+ preterm	73 (1.4%)	11 (2.0%)	54 (1.3%)
Multiple status, *N (%)*			
Singleton	5,033 (94.7%)	506 (93.0%)	3,910 (93.9%)
Twins or higher order	282 (5.3%)	38 (7.0%)	253 (6.1%)
Mode of delivery, *N (%)*			

© 2022 Cusimano MC et al. *JAMA Network Open.*

Spontaneous vaginal	3,131 (58.9%)	316 (58.1%)	2,369 (56.9%)
Operative vaginal	644 (12.1%)	44 (8.1%)	493 (11.8%)
Caesarean section	1,540 (29.0%)	184 (33.8%)	1,301 (31.3%)

© 2022 Cusimano MC et al. *JAMA Network Open.*
